# Food Insufficiency, Supplemental Nutrition Assistance Program (SNAP) Status, and 9-Year Trajectory of Cognitive Function in Older Adults: The Longitudinal National Health and Aging Trends Study, 2012–2020

**DOI:** 10.1016/j.tjnut.2022.12.012

**Published:** 2022-12-27

**Authors:** Muzi Na, Nan Dou, Monique J. Brown, Lenis P. Chen-Edinboro, Loretta R. Anderson, Alexandra Wennberg

**Affiliations:** 1 Department of Nutritional Sciences, Penn State College of Health and Human Development, University Park, PA, USA; 2 Department of Epidemiology and Biostatistics, Arnold School of Public Health, University of South Carolina, Columbia, SC, USA; 3 South Carolina SmartState Center for Healthcare Quality, Arnold School of Public Health, University of South Carolina, Columbia, SC, USA; 4 Rural and Minority Health Research Center, Arnold School of Public Health, University of South Carolina, Columbia, SC, USA; 5 Office for the Study on Aging, Arnold School of Public Health, University of South Carolina, Columbia, SC, USA; 6 Public Health Program, School of Health and Applied Human Sciences, University of North Carolina Wilmington, Wilmington, NC, USA; 7 Division of Gerontology, Department of Epidemiology & Public Health, University of Maryland School of Medicine, Baltimore, MD, USA; 8 Unit of Epidemiology, Institute of Environmental Medicine, Karolinska Institutet, Stockholm, Sweden

**Keywords:** food insecurity, food assistance programs, cognition, seniors, NHATS

## Abstract

**Background:**

Despite findings from cross-sectional studies, how food insecurity experience/Supplemental Nutrition Assistance Program (SNAP) status relates to cognitive decline over time has not been fully understood.

**Objectives:**

We aimed to investigate the longitudinal associations between food insecurity/SNAP status and cognitive function in older adults (≥65 y).

**Methods:**

Longitudinal data from the National Health and Aging Trends Study 2012–2020 were analyzed (n = 4578, median follow-up years = 5 y). Participants reported food insecurity experience (5-item) and were classified as food sufficient (FS, no affirmative answer) and food insufficient (FI, any affirmative answer). The SNAP status was defined as SNAP participants, SNAP eligible nonparticipants (≤200% Federal Poverty Line, FPL), and SNAP ineligible nonparticipants (>200% FPL). Cognitive function was measured via validated tests in 3 domains, and the standardized domain-specific and combined cognitive function z-scores were calculated. Mixed-effect models with a random intercept were used to study how FI or SNAP status was associated with combined and domain-specific cognitive z-scores over time, adjusting for static and time-varying covariates.

**Results:**

At baseline, 96.3% of the participants were FS and 3.7% were FI. In a subsample (n = 2832), 10.8% were SNAP participants, 30.7% were SNAP eligible nonparticipants, and 58.6% were SNAP ineligible nonparticipants. Compared with the FS group in the adjusted model (FI vs. FS), FI was associated with faster decline in the combined cognitive function scores [−0.043 (−0.055, −0.032) vs. −0.033 (−0.035, −0.031) z-scores per year, *P*-interaction = 0.064]. Cognitive decline rates (z-scores per year) in the combined score were similar in SNAP participants (β = −0.030; 95% CI: −0.038, −0.022) and SNAP ineligible nonparticipants (β = −0.028; 95% CI: −0.032, −0.024), both of which were slower than the rate in SNAP eligible nonparticipants (β = −0.043; 95% CI: −0.048, −0.038; *P*-interaction < 0.0001).

**Conclusions:**

Food sufficiency and SNAP participation may be protective factors preventing accelerated cognitive decline in older adults.

## Introduction

Food insecurity is a multidimensional phenomenon describing the circumstances in which people do not have adequate physical, social, or economic access to sufficient, safe, and nutritious food that meets their dietary needs for an active and healthy life [1]. Food insecurity persists among older adults aged 60 y and older in the United States. Prevalence of food insecurity due to financial resources has more than doubled and has increased from 5.5% to 12.4% in older adults 60 y or older in the past decade, according to an analysis based on the 2007–2016 National Health and Nutrition Examination Survey data [2]. Older adults living with food insecurity are more likely to suffer malnutrition [3], depression [4], and physical functioning limitations [5, 6]. Food insecurity is associated with the development of many chronic diseases that are prevalent in older populations, including diabetes, obesity, and cardiovascular diseases [[7], [8], [9], [10]].

Food insecurity may also accelerate age-related cognitive decline. A recent systematic review suggests that greater food insecurity experienced in later life is associated with reduced global cognitive function, executive function, and memory among middle-aged and older adults [11]. Only 2 longitudinal studies were identified, including 1 in Boston [12] and the other in the Chicago area [13]. Another longitudinal study published more recently was conducted in Europe and found that food insecurity experienced in early life, in contrast to food insecurity experience in later life, was associated with cognitive decline [14]. However, existing evidence is still sparse, including mostly cross-sectional findings in the review, which does not provide insights about the direction of the relationship between food insecurity experienced in later adulthood and poorer cognitive function or the trajectory of cognitive decline by food insecurity status in older adults.

Food insecurity is a major social justice issue in the older population. The “cycle of food insecurity and chronic disease” model suggests that there is a diet-dependent pathway linking food insecurity and subsequent changes in health status [15], potentially including adverse mental health outcomes. For example, dementia prevalence has been reported 14 percentage points higher in food insecure older adults than food secure peers [16]. The Supplemental Nutrition Assistance Program (SNAP) is the largest federally funded nutrition assistance program in the United States, which is known to reduce hunger and food insecurity in the general population [[17], [18], [19]]. However, little translational evidence is available on how food insecurity or SNAP status may impact brain aging in older adults. The goal of this study was to investigate the relationship between food insecurity experience, SNAP status, and subsequent cognitive decline in a nationally representative sample of older adults in the United States.

## Methods

### Study design and participants

Data used in this study are from the National Health and Aging Trends Study (NHATS), a nationally representative survey of Medicare beneficiaries aged 65 y and older living in the United States [20]. The survey was conducted under a complex survey sampling design and oversampled individuals over 90 y old and non-Hispanic Black individuals [20]. In 2011, NHATS recruited 8245 eligible participants and followed them annually to collect information about sociodemographic status; social, physical, and technological environment; medical comorbidities; and cognitive function. To be included in the current study, participants needed to have completed data for the food insecurity experience and cognitive assessment data for ≥2 survey rounds. To prevent response bias on cognitive outcomes, participants were excluded if they had probable dementia in any survey year, which was determined by self-reported dementia diagnosis or a score of ≥2 on the AD8 Dementia Screening Interview [21]. This study used deidentified NHATS data, which met the definition for non-human subject research; therefore, it was exempt from Institutional Review Board review.

### Measures

#### Food insecurity experience and food insufficiency classification

Participants were asked annually to report the individual-level food insecurity experience due to financial, social, and functional limitations based on 5 questions. The 5 questions inquired whether or not the participant had experienced the following in the past 30 d: *1*) “going without groceries”; *2*) “going without hot meals”; *3*) “going without eating due to lack of ability”; *4*) “going without eating because of lack of social support to do so”; and *5*) “skipping meals due to financial constraints.” Following published methodology by Tucher et al. [22], the number of affirmative answers was calculated as the summary indicator of food insecurity, which showed associations with a wide range of demographic and biopsychosocial factors in NHATS. Participants with the summary indicator of 0 were classified as food sufficient (FS) and those with summary indicator of ≥1 were classified as food insufficient (FI).

#### SNAP status

Participants were asked “In the last year, did you receive help from food stamps?” SNAP participants were defined if they provided an affirmative response to the survey question and nonparticipants were otherwise defined. Total household income and household size data were used to calculate the proportion of people falling at or below the 200% Federal Poverty Line (FPL), which was used to define SNAP (formally called food stamp program) eligibility in the elderly [23]. SNAP participation and eligibility data were available in 2832 participants. Among the nonparticipants with eligibility information, we further categorized them as SNAP eligible nonparticipants (≤200% FPL) or SNAP ineligible nonparticipants (>200% FPL).

#### Cognitive function

Participants’ cognitive function was objectively assessed annually in 3 domains: memory, orientation, and executive function. To assess memory, participants were asked to complete a 10-item word-list memory task from the Consortium to Establish a Registry for Alzheimer’s Disease neuropsychological battery [24]. They were asked to recall the words immediately after being presented (that is, immediate recall), and again 5 min after the word presentation (that is, delayed recall). The memory score was calculated as total number of words recalled correctly during the immediate and delayed recall tasks, ranging from 0 to 20. The orientation test required participants to provide the date, month, year, and day of the week of the interview date, as well as the first and last names of the current President and Vice-President of the United States. One point was assigned to each correct answer, yielding a total orientation score that possibly ranged from 0 to 8. The Clock Drawing Test was applied to assess executive function [25, 26]. Participants were asked to draw a clock face with the time as “10 after 11” on a given sheet within 120 s. Based on the standard criteria, their drawing tests were scored on a scale ranging from 0 to 5, in which 0 = “not recognizable as a clock,” 1 = “severely distorted depiction of a clock,” 2 = “moderately distorted depiction of a clock,” 3 = “mildly distorted depiction of a clock,” 4 = “reasonably accurate depiction of a clock,” and 5 = “accurate depiction of a clock.” All individual test scores were standardized into z-scores for comparability, and the mean of the domain-specific z-scores was calculated as a combined cognitive function z-score. Higher z-scores in memory, orientation, executive function, and combined cognitive function indicated better cognitive function among older adults.

### Covariates

The following baseline sociodemographic information collected in the 2012 NHATS were included in the model estimation: age group (65–69, 70–74, 75–79, 89–84, 85–89, and ≥90 y), sex (male or female), race/ethnicity (White, non-Hispanic; Black, non-Hispanic; Hispanic; and other), education levels (less than high school, high school, and post–high school education), and total income (in quintiles). All of the sociodemographic variables were collected annually, except for total income, which was collected biennially since 2011. Because these variables do not vary over time (for example, income quintile) or vary over time uniformly (for example, age), we have included the baseline data of these variables for adjustment.

Data on time-varying covariates were collected at the time of each cognitive assessment annually. The time-varying covariates included marital status (“married or living with partner” or “not married, widowed, separated, or divorced”), BMI (kg/m^2^, calculated based on the self-reported weight/self-reported height^2^), depressive symptoms score [calculated based on the validated 2-item Patient Health Questionnaire [27]; range, 0–6], anxiety score [calculated based on the validated 2-item Generalized Anxiety Disorder Scale [28]; range, 0–6], and self-reported diagnosed chronic conditions, including hypertension (yes/no), diabetes (yes/no), heart disease (yes/no), and heart attack or myocardial infarction (yes/no).

### Statistical analysis

After adjusting for complex sampling design, the weighted means (SE) and proportions describing sociodemographic characteristics, nutritional status, and health conditions at baseline were calculated in the overall sample and by baseline FI status. To test the differences of weighted means by baseline FI status or by SNAP status, linear regressions of continuous variables were performed as a function of the categorical FI status, adjusting for survey data. The adjusted Wald tests were used to examine the equivalence of weighted means in each group. For categorical variables, chi-square tests were performed using survey data to compare the weighted proportions in each group.

Mixed-effect models without accounting for sampling weights were used to examine how FI status was associated with the combined and domain-specific cognitive z-scores over time. The random intercept was specified in the models to adjust for within-subject effects, and the categorical-by-continuous interaction term between FI status and year was included to estimate and compare cognitive decline in the 2 groups. Multiple covariates were included in the model, including the baseline sociodemographic variables (age, sex, race/ethnicity, education levels, and income quintiles) and the annually collected time-varying variables (marital status, BMI, depression and anxiety score, and health conditions). We adjusted for the time-varying variables to account for updated information in the model estimation. The command “margins” was analyzed after the adjusted mixed-effect models and the “marginsplot” function was used to estimate the adjusted slope of cognitive decline in each group. To better interpret the clinical significance of our results, we calculated the estimate of equivalent age difference in years that corresponded to the difference in cognitive decline rates between the FI and FS older adults, using a previously published method [29].

To examine if cognitive decline rates differ by SNAP status, in the subsample for which data was available (n = 2832), we performed the analysis among the SNAP participants, SNAP eligible nonparticipants (≤200% FPL), and SNAP ineligible nonparticipants (>200% FPL) and tested the interaction between SNAP status and year in similarly adjusted mixed-effect models.

Several sensitivity analyses were performed, and results are presented in the Supplementary Material. We additionally adjusted for baseline cognitive function score but chose to present the more conservative findings from the models without adjustment for baseline cognitive function as main results, because baseline-adjusted models may introduce a spurious statistical association [30]. Educational attainment is a leading factor in promoting cognitive reserve [31]. The “cumulative advantage-disadvantage theory” hypothesizes that education-related disparities and inequality originated in early childhood may continue to be amplified through the life course [32], suggesting that educational attainment may attenuate aging-associated cognitive declines across the life span [33]. Therefore, additional stratification analysis by educational attainment was performed to further examine the associations between food insecurity experience and cognitive decline by educational levels. To test the role of education level as a potential effect modifier, a 3-way multiplicative term between FI status, year, and education attainment was computed and tested in the adjusted models of the combined cognitive function score. The sample size was too small for the stratification analysis by education level and by SNAP status in the subsample.

All statistical analyses were performed using Stata/SE, version 17.0 (StataCorp).

## Results

Based on inclusion and exclusion criteria (Supplemental Figure 1), there were 4578 participants from NHATS included in the analytic sample, representing a population size of 22,060,979 older adults. The number of completed annual cognitive assessments ranged from 2 to 9, with a median (IQR) of 6 [3, 9] assessments, and 61.6% of the included participants had 5 or more cognitive assessments. In the subsample of 2832 participants in which SNAP status information was available, the median (IQR) number of cognitive assessments was 7 [3, 9].

Participants’ characteristics at baseline are presented in Table 1. In our sample, 4400 (96.3%) of the participants were categorized as FS and 178 (3.7%) were FI. Age distribution by the 5-y age categories did not differ by baseline FI status (*P* = 0.187). Compared with the FS group, more FI older adults were females, racial/ethnic minorities, had lower educational attainment, were not married, and had lower household income (all *P* < 0.005). The FI participants also had higher proportions of being eligible for SNAP, receiving food stamps, and other food assistance programs (all *P* < 0.001). In terms of the nutrition and health status, the mean depression (*P* < 0.001) and anxiety scores (*P* < 0.001) were higher among the FI participants. Compared with the FS group, there was a higher proportion of underweight and obesity (*P* = 0.004), hypertension (*P* = 0.063), diabetes (*P* = 0.003), heart diseases (*P* = 0.001), and heart attack or myocardial infarction (*P* = 0.041) among the FI participants.

**TABLE 1:** Sample characteristics of the NHATS participants at baseline by food sufficiency status and by SNAP status^1^

	Overall sample	All	Food sufficiency status^2^			Subsample	All	SNAP status^3^			
			Food sufficient	Food insufficient	*P*			SNAP participants	SNAP eligible nonparticipants (≤200% FPL)	SNAP ineligible nonparticipants (>200% FPL)	*P*
N	4578		4400	178		2832		379	993	1460	
%			96.3	3.7				10.8	30.7	58.6	
Age groups, %	4578				0.187	2832					<0.001
65–69 y		29.6	29.8	23.9			23.3	35.2	32.6	23.3	
70–74 y		26.1	26.1	25.6			26.4	26.9	30.8	26.4	
75–79 y		18.7	18.8	17.7			21.0	18.2	18.2	21.0	
80–84 y		14.5	14.4	16.4			15.8	12.3	11.1	15.8	
85–89 y		8.0	7.9	10.8			10.2	5.5	5.5	10.2	
≥90 y		3.1	3.0	5.6			3.3	2.0	1.7	3.3	
Females, %	4578	56.4	68.8	56.4	0.004	2832	51.1	62.3	60.6	44.0	<0.001
Race/Ethnicity, %	4548				<0.001	2826					<0.001
White, non-Hispanic		84.8	85.0	77.3			83.5	55.3	76.3	92.5	
Black, non-Hispanic		7.4	7.4	8.1			7.7	20.9	10.9	3.6	
Hispanic		2.5	2.5	1.5			2.5	4.4	3.5	1.6	
Other		5.4	5.1	13.1			6.3	19.4	9.4	2.3	
Education, %	4554				<0.001	2831					<0.001
<High school diploma		18.0	17.4	32.2			18.4	53.9	28.4	6.7	
High school diploma		34.4	34.4	35.1			33.5	31.9	42.8	28.9	
>High school diploma		47.6	48.2	32.8			48.1	14.2	28.8	64.4	
Marital status, %	4572				<0.001	2830					<0.001
Married, living with partner		58.1	59.2	29.0			56.7	26.6	39.3	71.4	
Not married, widowed, separated, divorced		41.9	40.8	71.0			43.3	73.4	60.8	28.6	
Household income quintiles, %	4578				<0.001	2832					<0.001
1 ($0–$9,000)		9.5	9.0	23.2			9.9	38.2	18.8	0.0	
2 ($9001–$17,402)		15.7	15.1	30.0			15.9	47.2	35.3	0.0	
3 ($17,403–$30,000)		21.8	21.7	24.7			20.6	10.8	40.6	11.9	
4 ($30,001–$55,000)		22.6	22.9	12.9			22.4	1.5	5.3	35.2	
5 (>$55,000)		30.5	31.4	9.2			31.2	2.4	0.0	52.9	
BMI, kg/m^2^	4468	27.7 (0.10)	27.7 (0.11)	28.3 (0.51)	0.276	2768	27.9 (0.13)	29.1 (0.43)	28.4 (0.24)	27.5 (0.18)	<0.001
BMI classification, %	4468				0.004	2791					<0.001
Underweight (<18.5)		1.9	1.7	5.4			1.5	1.8	2.7	0.9	
Normal (18.5–24.9)		31.3	31.6	23.2			29.8	28.9	27.8	31.0	
Overweight (25.0–29.9)		38.4	38.5	35.8			37.7	30.9	33.4	41.1	
Obesity (≥ 30.0)		28.4	28.1	35.5			31.0	38.4	36.1	27.0	
Depression, score	4548	2.8 (0.03)	2.7 (0.02)	4.2 (0.14)	<0.001	2816	2.8 (0.03)	3.5 (0.09)	3.1 (0.05)	2.6 (0.04)	<0.001
Anxiety, score	4562	2.8 (0.02)	2.7 (0.02)	4.1 (0.15)	<0.001	2823	2.8 (0.03)	3.4 (0.09)	3.0 (0.05)	2.6 (0.03)	<0.001
Hypertension, %	4576	65.8	65.5	73.3	0.063	2830	66.6	80.9	69.7	62.4	<0.001
Diabetes, %	4578	24.3	23.9	34.2	0.003	2832	25.1	39.4	28.2	20.9	<0.001
Heart diseases, %	4576	19.1	18.7	29.7	0.001	2830	19.3	25.4	21.4	17.0	<0.001
Heart attack or myocardial infarction, %	4576	2.6	2.5	5.1	0.041	2831	2.4	3.8	2.2	2.3	0.216

SNAP, Supplemental Nutrition Assistance Program.

1 Values are mean (SE) or % after adjusting for complex sampling scheme. Depressive symptoms score was calculated based on the validated 2-item Patient Health Questionnaire [22]. Anxiety score was calculated based on the validated 2-item Generalized Anxiety Disorder Scale [23].

2 Food sufficiency status was defined using a summary indicator of food insecurity calculated based on 5-item questions [18], as food sufficient if summary indicator was 0 and food insufficient if summary indicator was >0.

3 SNAP eligibility is defined as ≤200% Federal Poverty Level (FPL) for households with older adults. SNAP participation and eligibility data were available in 2832 participants.

In the subsample, the number (%) of SNAP participants, SNAP eligible nonparticipants (≤200% FPL), and SNAP ineligible nonparticipants (>200% FPL) were 379 (10.8%), 993 (30.7%), and 1460 (58.6%), respectively. More SNAP participants and SNAP eligible nonparticipants, as compared with the SNAP ineligible nonparticipants, were in the younger age groups, females, racial and ethnic minorities, not married, widowed, separated or divorced, and in the lowest 2 income quintiles (all *P* < 0.001). Across the 3 SNAP status groups, mean BMI, depression score, anxiety score, and the proportions of underweight, obesity, and diagnosed chronic conditions (hypertension, diabetes, and heart disease) decreased from SNAP participants, to SNAP eligible nonparticipants, to those who were SNAP ineligible, and the differences by SNAP status were significant (all *P* < 0.001).

**FIGURE 1:**
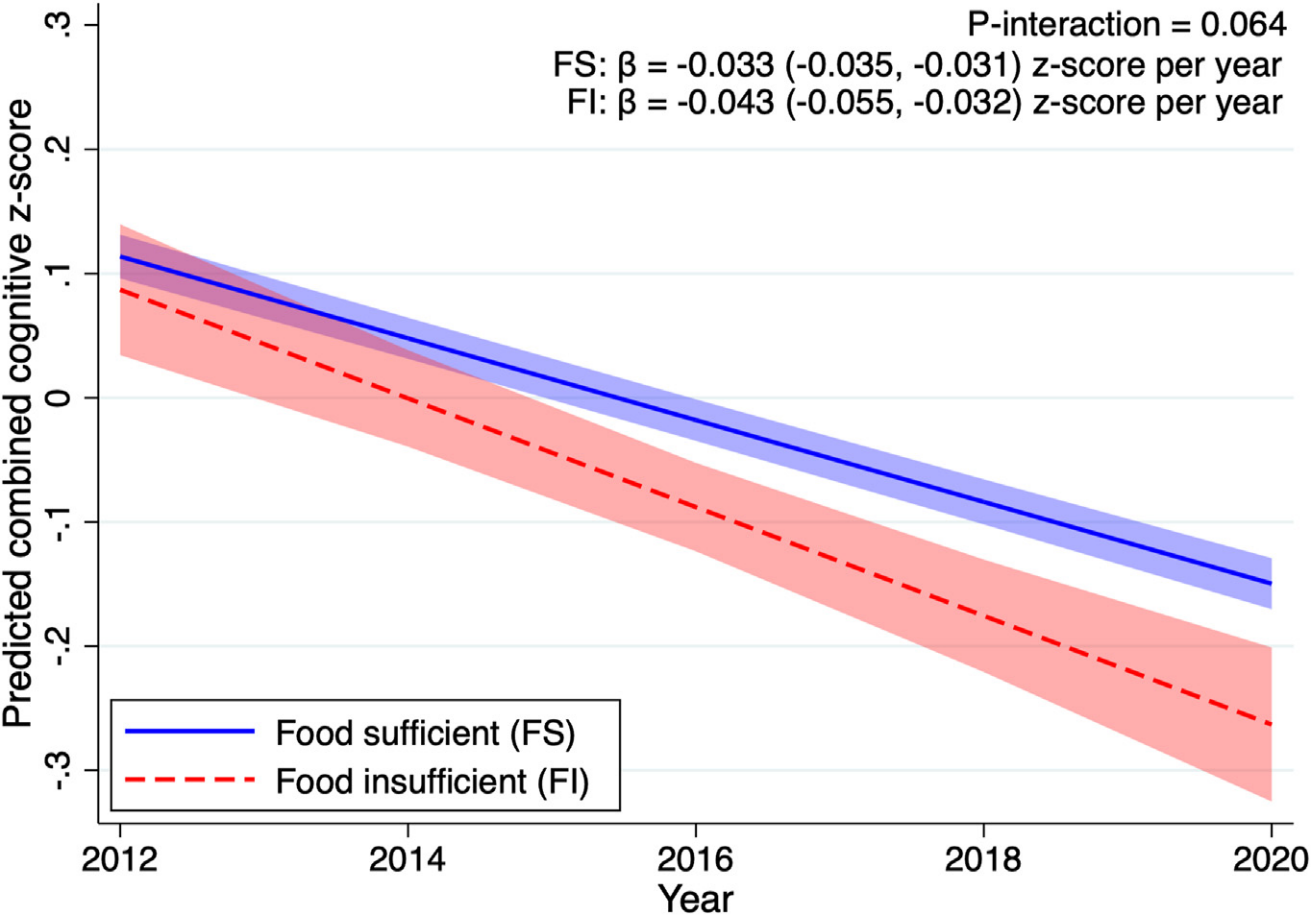
The predicted trajectory of combined cognitive function z-score between 2012 and 2020 in older adults by food insufficiency status (n = 4578). Lines represent the estimated trajectory of combined cognitive score and shaded areas represent the 95% CI. The cognitive decline rates were modeled based on the mixed model with the categorical food insufficiency by year interaction term, adjusting for baseline age, sex, race/ethnicity, education levels, income quintiles, and time-varying variables collected annually for marital status, BMI, depression score, anxiety score, diagnosed status for hypertension, diabetes, heart disease, and heart attack or myocardial infarction. *P*-interaction was tested at a significance level of 0.05.

**FIGURE 2:**
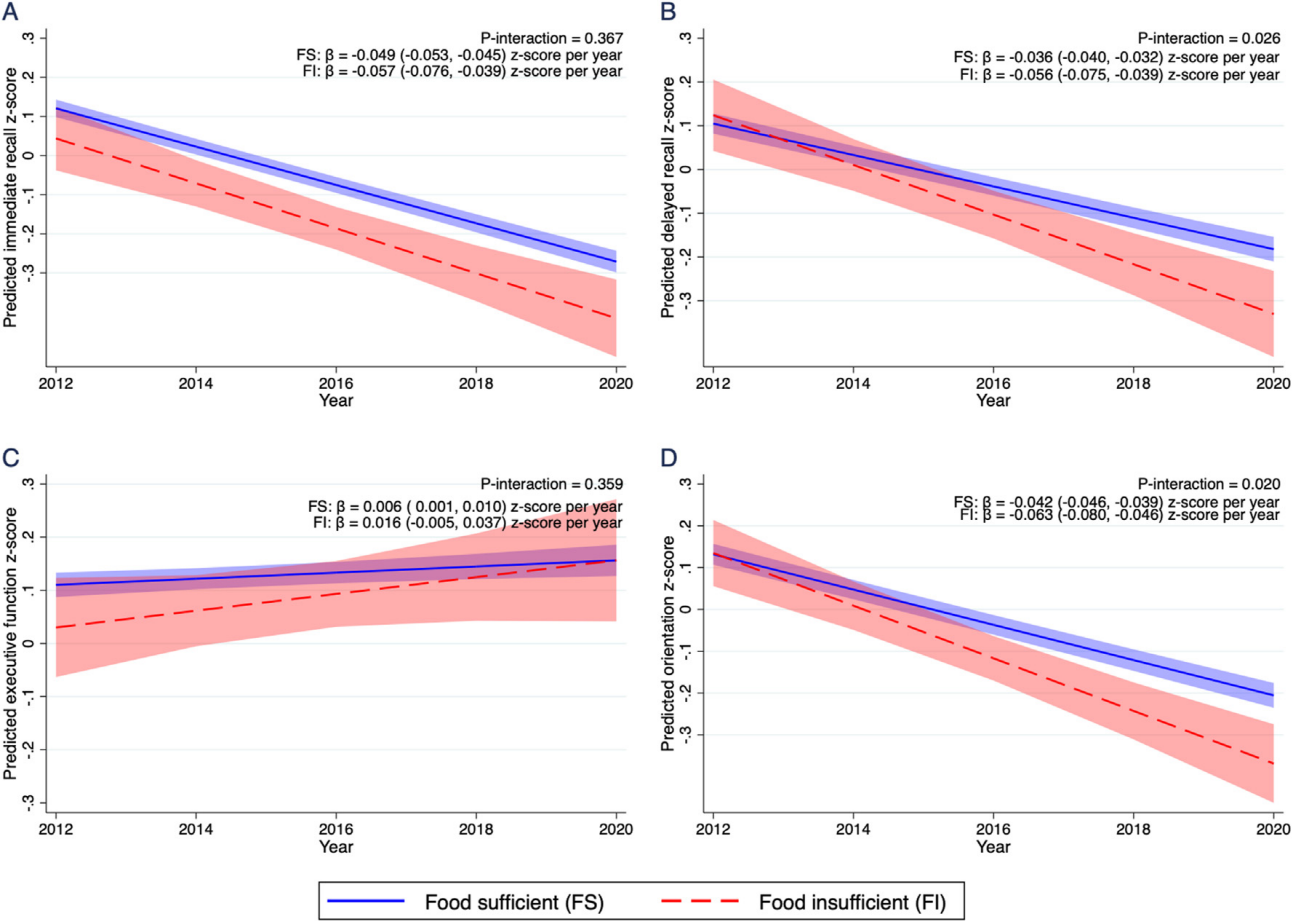
The predicted trajectory of (A) immediate recall z-score, (B) delayed recall z-score, (C) executive function z-score, and (D) orientation z-score between 2012 and 2020 in older adults by baseline food insufficiency status (n = 4578). Lines represent the estimated trajectory of combined cognitive score and shaded areas represent the 95% CI. The cognitive decline rates were modeled based on the mixed model with the categorical food insufficiency by year interaction term, adjusting for baseline age, sex, race/ethnicity, education levels, income quintiles, and time-varying variables collected annually for marital status, BMI, depression score, anxiety score, diagnosed status for hypertension, diabetes, heart disease, and heart attack or myocardial infarction. *P*-interaction was tested at a significance level of 0.05.

The estimated trajectories of combined cognitive function and domain-specific cognitive function z-scores by FI status are shown in FIGURE 1, FIGURE 2, respectively. In the adjusted model, baseline combined cognitive function z-scores did not differ by FI status (β = −0.005; 95% CI: −0.076, 0.066 z-scores, *P* = 0.888). Significant declines of the combined cognitive function z-scores over time were observed in both the FS and FI groups. There was a marginally statistically significant modifying association of FI status on the slope of decline (*P*-interaction = 0.064), in which we saw a faster decline in the FI older adults (β = −0.043; 95% CI: −0.055, −0.032 z-scores per year), compared with the FS group (β = −0.033; 95% CI: −0.035, −0.031 z-scores per year). The greater cognitive decline rate observed in the FI group was equivalent to being 3.8 y older. Findings were similar in the model that additionally adjusted for the combined cognitive function z-scores at baseline (Supplemental Figure 2, *P*-interaction = 0.016).

At baseline, the mean immediate recall and executive function z-scores were lower in the FI group as compared with the FS group; however, the differences were not statistically significant (*P* = 0.298 for immediate recall z-score; *P* = 0.130 for the executive function z-score). When examining the trajectory of domain-specific cognitive function in relation to FI status, we observed a significant decline in both the FS and FI groups in immediate recall z-score (Figure 2A), delayed recall z-score (Figure 2B), and orientation z-score (Figure 2D), but not in executive function z-score (Figure 2C). Specifically, adults who were FI had statistically significant faster annual decline of 0.056 (95% CI: −0.075, −0.039) z-scores in the delayed recall domain, as compared with the FS group, whose decline rate was 0.036 z-scores per year (95% CI: −0.040, −0.032; *P*-interaction = 0.026). For the orientation domain, the slope of decline was also significantly greater in the FI group (β = −0.063; 95% CI: −0.080, −0.046 z-scores per year) as compared with the FS group (β = −0.042; 95% CI: −0.046, −0.039 z-scores per year, *P*-interaction = 0.020). Additionally adjusting for baseline cognitive test scores yielded consistent results with slightly steeper estimated slope and comparable or smaller *P*-interactions (Supplemental Figure 3).

**FIGURE 3:**
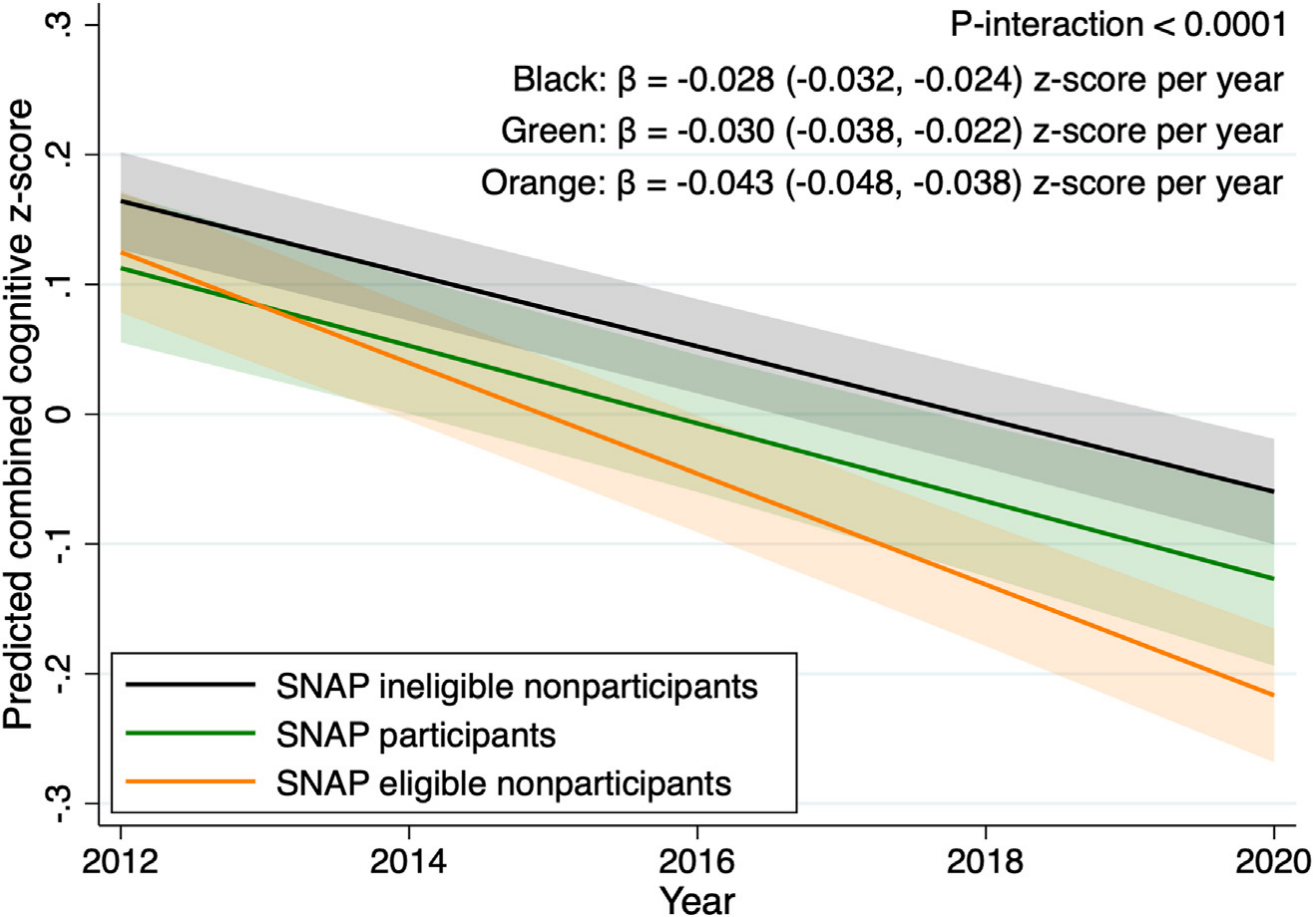
The predicted trajectory of combined cognitive function z-score between 2012 and 2020 in older adults by SNAP status (n = 2832). Lines represent the estimated trajectory of combined cognitive score and shaded areas represent the 95% CI. The cognitive decline rates were modeled based on the mixed model with the categorical food stamp/SNAP participation and eligibility by year interaction term, adjusting for baseline age, sex, race/ethnicity, education levels, income quintiles, and time-varying variables collected annually for marital status, BMI, depression score, anxiety score, diagnosed status for hypertension, diabetes, heart disease, and heart attack or myocardial infarction. *P*-interaction was <0.0001. SNAP, Supplemental Nutrition Assistance Program.

**FIGURE 4:**
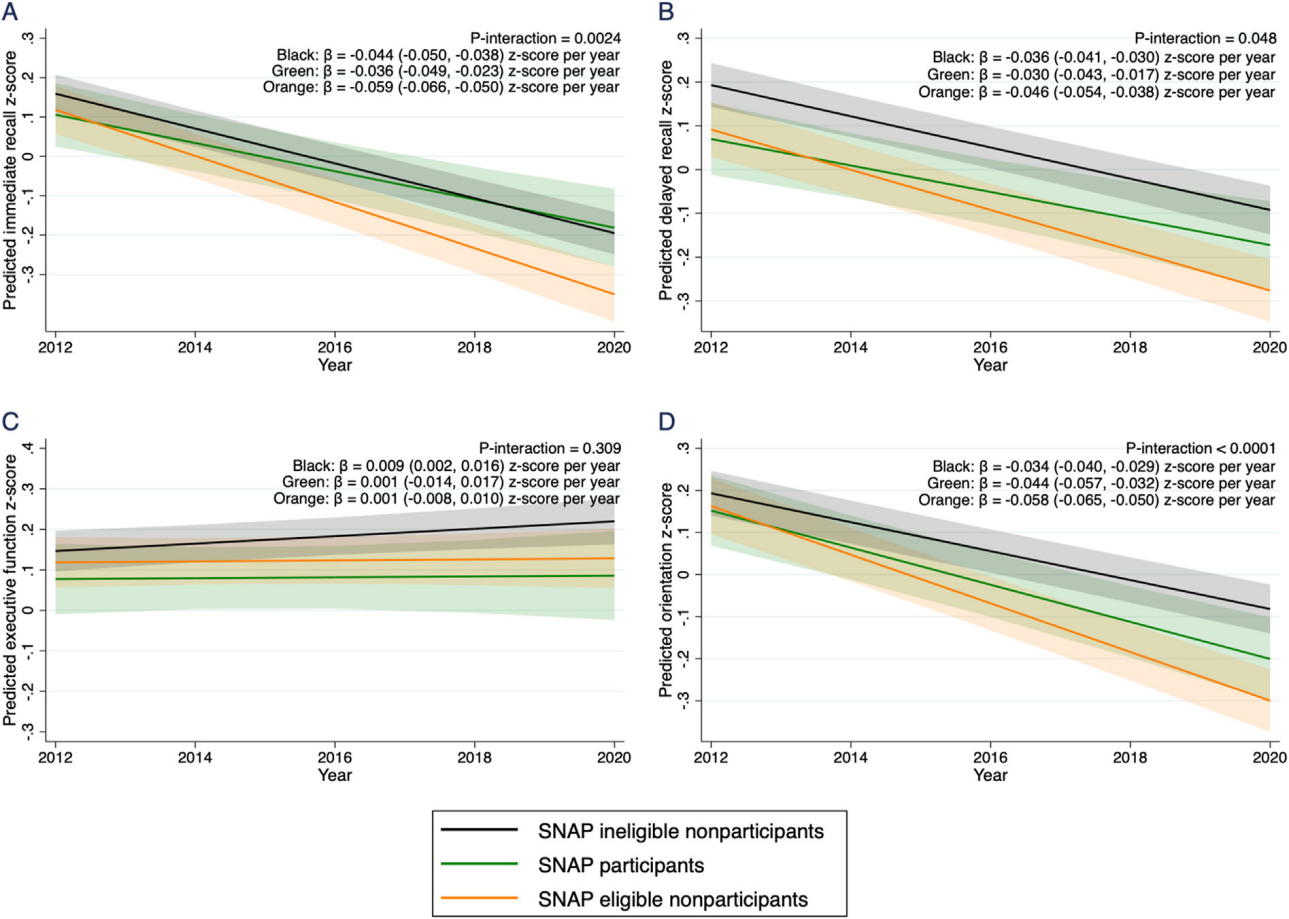
The predicted trajectory of (A) immediate recall z-score, (B) delayed recall z-score, (C) executive function z-score, and (D) orientation z-score between 2012 and 2020 in older adults by SNAP status (n = 2832). Lines represent the estimated trajectory of combined cognitive score and shaded areas represent the 95% CI. The cognitive decline rates were modeled based on the mixed model with the categorical food insufficiency by year interaction term, adjusting for baseline age, sex, race/ethnicity, education levels, income quintiles, and time-varying variables collected annually for marital status, BMI, depression score, anxiety score, diagnosed status for hypertension, diabetes, heart disease, and heart attack or myocardial infarction. *P*-interaction was tested at a significance level of 0.05. SNAP, Supplemental Nutrition Assistance Program.

In a subsample in which SNAP status information was available (n = 2832), the SNAP eligible nonparticipants (≤200% FPL) had the fastest cognitive decline rate (β = −0.043; 95% CI: −0.048, −0.038 z-scores per year) than the rate observed in SNAP participants (β = −0.030, 95% CI: −0.038, −0.022 z-scores per year) and SNAP ineligible nonparticipants (>200% FPL) (β = −0.028, 95% CI: −0.032, −0.024 z-scores per year) (Figure 3, *P*-interaction < 0.0001). The faster cognitive decline rate observed in the SNAP eligible nonparticipants as compared with SNAP participants was equivalent to being 4.5 y older. Significant interactions were also observed in the domain-specific cognitive function z-scores (Figure 4) for immediate recall (*P*-interaction = 0.002), delayed recall (*P*-interaction = 0.048), and orientation (*P*-interaction < 0.001), in which SNAP eligible nonparticipants had the fastest decline rate as compared with the other 2 groups. For the combined (Supplemental Figure 4) and all of the domain-specific cognitive function z-scores (Supplemental Figure 5), additionally adjusting for the corresponding baseline cognitive function z-scores did not alter the findings.

When investigating the association between FI status and cognitive decline stratified by participants’ education level, there was a dose-response relationship between the educational attainment level and the baseline combined cognitive function score. Using less than high school education as the reference group, the baseline combined score was 0.23 higher (95% CI: 0.17, 0.28) in high school graduates and 0.39 higher (95% CI: 0.34, 0.45) in the greater than high school education level group. However, there was no evidence that education modified the association between FI status and cognition (Supplemental Figure 6, *P*-interaction = 0.695).

## Discussion

In this national sample of older adults 65 y or older, we have identified different trajectories of cognitive decline between 2012–2020 by their food insufficiency status or their SNAP status. After controlling for baseline and time-varying socioeconomic characteristics, BMI, mental health, and medical conditions, the FI group had marginally faster combined cognitive function score decline and significantly faster delayed recall and orientation decline over the 9-y span. In a subsample, SNAP participants had comparable cognitive decline rates with SNAP ineligible nonparticipants in the combined and 3 out of 4 of the domain-specific cognitive function scores, whereas SNAP eligible nonparticipants had the fastest decline rates. All of the observed trajectory of cognitive decline was consistent when additionally adjusted for the corresponding cognitive function scores at baseline. Despite the differences in cognitive scores at baseline, education level in the overall sample did not seem to modify the observed relationship between FI and cognitive decline. Independent of several sociodemographic, nutrition, and health status variables, our study showed that food insecurity experience in late life were associated with faster cognitive decline, whereas SNAP participation was associated with slower cognitive decline as compared with the SNAP eligible nonparticipants. As assessed by the combined cognitive score changes, the FI-associated decline was equivalent to being 3.8 y older and the SNAP eligible nonparticipants’ decline, as compared with the SNAP participants, was equivalent to being 4.5 y older.

Our findings from this longitudinal study are in line with a recent systematic review of food insecurity and cognitive function, in which food insecurity experience in late life was adversely associated with global cognitive function and specific cognitive domains [11]. According to a systematic review of 170 studies that examined the associations between food insecurity and dietary quality, food insecurity was consistently associated with poorer dietary quality primarily characterized by lower consumption of vegetables, fruits, and dairy products [34]. Vitamins (for example, B group, E), folate, and flavonoids and plant-rich dietary patterns are thought to have antioxidant and anti-inflammatory properties that could relate to the observed neuroprotective association in our study. However, evidence seems to be more consistent for the benefits of an overall dietary pattern, such as the Mediterranean diet [35, 36] and the Mediterranean-Dietary Approaches to Stop Hypertension Intervention for Neurodegenerative Delay diet [37], as compared with individual food groups or nutrients [38, 39]. In addition, food insecurity experiences increase stress levels [4], which may in turn predict faster cognitive decline [40].

Through repeated suboptimal food intake and increased stress, food insecurity may cause physiological changes that further lead to cognitive decline. Allostatic load is a measure of the cumulative physiological wear-and-tear reflected in the primary system as neuroendocrine and/or inflammatory disturbances and the secondary system as metabolic and cardiovascular disturbances. In a sample of Puerto Ricans adults aged 45–75 y (n = 733), food insecurity was associated with high primary system scores [41]. Similar results were seen in a large nationally representative sample of US adults aged ≥50 y (n = 14,394), in which food insecurity was associated with elevated inflammatory, cardiovascular, and other metabolic responses [42]. Inflammation and metabolic alterations, independently [43] and interactively [44], may be important mechanisms underlying cognitive impairment and dementia in older adults. Future studies are required to investigate how food insecurity experience impacts the primary and secondary systems specifically and the implications of this impact on brain health.

The analysis by SNAP status showed fastest cognitive decline in the SNAP eligible nonparticipants, as compared with the SNAP participants and SNAP ineligible nonparticipants. Previous evaluation studies have shown that SNAP may have both nutritional and nonnutritional benefits, including improvement of food security [[17], [18], [19]], promotion of mental health among food insecure general adults [45] and older adults [46], and promotion of general health [47]. These benefits may translate to the observed difference in cognitive decline rates by SNAP status; however, the underlying mechanisms remain to be studied. Given the low participation rate in the food assistance program in older adults, we were unable to further investigate what roles SNAP may play in food insecurity and cognitive decline in this NHATS sample. The findings, however, should be interpreted with caution. It is known that the characteristics at the individual and community level differ between participants and eligible nonparticipants [48, 49], many of which were not measured in NHATS. A prior study analyzing national panel data of the Health and Retirement Study found reduced levels of cognitive functioning were associated with reduction in SNAP uptake among eligible older adults aged 60 and older [50], indicating a potential bidirectional relationship between SNAP status and disparities in cognitive function.

Many of the sociodemographic, nutritional, and health characteristics in our sample differ by their food sufficiency or SNAP status. Disparities in food insecurity risk, SNAP participation, and cognitive function have been observed by race and ethnicity [[51], [52], [53], [54]], socioeconomic status [55, 56], nutritional status [[57], [58], [59]], and the presence of chronic conditions [10, 50, 60]. Adjusting for the potential confounders, we still observed faster trajectories of cognitive decline in FI older adults as compared with the FS referent group and among SNAP eligible nonparticipants as compared with the SNAP participants and SNAP ineligible nonparticipants. In our study, there was no statistical evidence of a differential association between food sufficiency status and the combined cognitive scores by education, despite the expected differences of cognitive function scores at baseline. This finding is consistent with a recent review of evidence that educational attainment was positively associated with levels of cognitive ability in adulthood (an advantage of 0.2–0.4 standard deviations in cognitive performance per every ≥5 y of education); however, the association between education and aging-associated cognitive declines was negligible (<0.008 standard deviations per decade) [33].

In NHATS, food insecurity experience was measured using a summary indicator of 5 questions that covered both financial and nonfinancial constraints of food access [22]. Additional obstacles for older adults to acquire food may include poor functional status, poor environmental context, and lack of social resources [61]. Although the indicator included broader challenges beyond financial limitations and showed some amount of external validity in relation to biopsychosocial factors [22], the summary indicator did not include all the possible financial related food insecurity experiences as compared with other commonly used food insecurity scales, such as the US Household Food Security Survey Module. Underestimation of food insecurity prevalence may have occurred in our study. The food insecurity prevalence in the NHATS sample of Medicare beneficiaries was ∼4%, which was lower than a previously reported prevalence of 7%–12% using the US Household Food Security Survey Module in 2011–2016 [2], but was similarly low in another study (5.7%) of predominantly (∼80%) Medicare Advantage beneficiaries [62]. In addition, using the binary indicator, we were not able to determine the different degrees of food insecurity experienced by older adults and their potential impact on cognitive decline.

There are other limitations of the study worth noting. Unmeasured confounding could have led to inaccurate estimates of the relationship between food insecurity status/SNAP status and cognitive decline. For example, NHATS did not collect data on physical activities. The only variables collected were a list of “favorite activities” without the measurement of intensity or duration; therefore, physical activity could not be controlled for in the models. Information on medication use was also unavailable and could confound the observed association because it could be associated with food insecurity status [63] and could alter cognitive functions [64, 65]. Objective measures of chronic conditions, such as trajectory of cardiovascular biomarkers, in addition to the chronic condition status, are potentially predictive of cognitive decline [66]. Biomarkers of these conditions were not available in NHATS. However, treating disease status as a time-varying covariate in our models took change of disease diagnosis over time into account. The lack of dietary data in NHATS prevented us from examining the potential mediating impact of suboptimal diet in the observed relationships. Finally, our analytic sample was predominantly non-Hispanic White (85%). About half of the sample had higher than a high school education and ∼3 in 10 of the original sample were in the highest income quintile. Future longitudinal studies should consider strategies to better retain racially/ethnically diverse subgroups and participants of lower socioeconomic status, who may have experienced food insecurity adversity throughout multiple life stages. Going beyond nationally representative samples, studies focused on vulnerable populations are warranted to better understand and address the disparities in food insecurity risk, SNAP participation, and mental health.

In conclusion, compared with the FS referent group, we have observed marginally faster cognitive decline, assessed by the combined cognitive score, and significantly faster decline in 2 out of 4 of the individual cognitive domains, over a 9-y follow-up among FI older adults in a national sample of adults ≥65 y old. In a subsample, the decline rate of SNAP eligible nonparticipants was significantly higher in the combined and 3 of 4 examined individual cognitive domains, as compared with the SNAP participants and the SNAP ineligible nonparticipants. The greater cognitive decline rate observed in the FI group was equivalent to being 3.8 y older, whereas the greater cognitive decline rate observed in the SNAP eligible nonparticipation group was equivalent to being 4.5 y older. With careful design considering ethical and feasibility considerations, future intervention studies are warranted to investigate the impact of addressing food insecurity and promoting SNAP participation on cognitive health in older adults.

## Funding

Supported by the Broadhurst Career Development Professorship for the Study of Health Promotion and Disease Prevention (MN) and the National Institute of Mental Health: K01MH115794 (MJB).

## Author disclosures

The authors report no conflicts of interest.

## Data Availability

Data described in the manuscript are publicly and freely available without restriction at the National Health and Aging Trends Study (https://www.nhats.org/researcher/data-access). The code book and analytic code will be made available upon request pending application to and approval by the corresponding author, Dr. Muzi Na.
