# Supplementary material for: Food Insufficiency, Supplemental Nutrition Assistance Program (SNAP) Status, and 9-Year Trajectory of Cognitive Function in Older Adults: The Longitudinal National Health and Aging Trends Study, 2012–2020
Source: J Nutr. 2022 Dec 27;153(1):312–21. doi: 10.1016/j.tjnut.2022.12.012 (PMC10196579; doi:10.1016/j.tjnut.2022.12.012)
Supplement: Multimedia component 1 [file mmc1.docx]

**Supplemental Materials**

Excluded participants with missing responses on covariates, N = 126

Excluded participants with missing responses on food insecurity status and use of food assistance programs at baseline, N = 963

Excluded participants with only one cognitive assessment from 2012-2020, N = 1,137

Participants enrolled at baseline (2012), N= 7,075

Study population, N= 4,451

Excluded participants who had probable dementia at baseline, N = 398

**Supplemental figure 1: Flow chart of the study population based on inclusion and exclusion criteria**

**
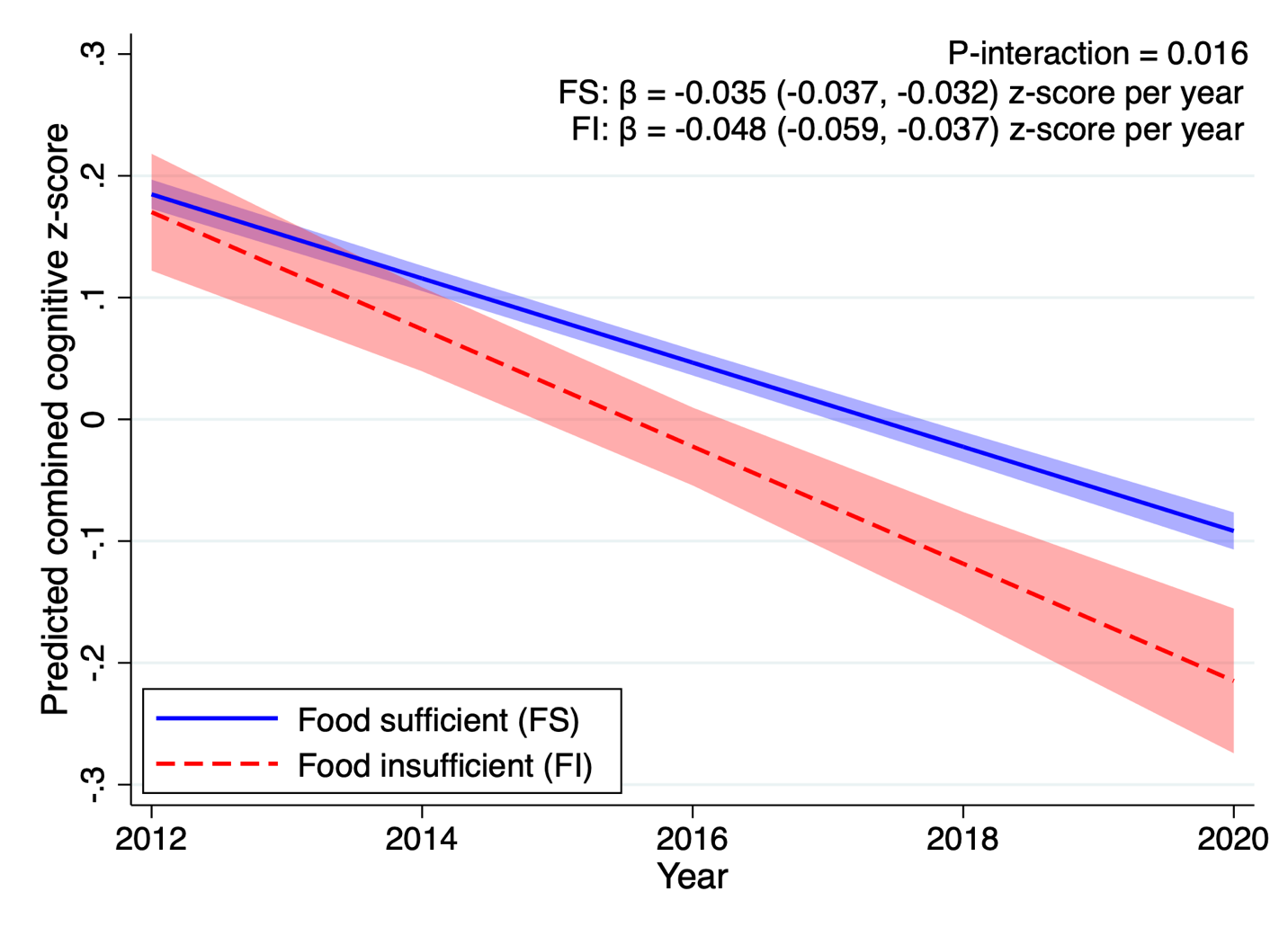
**

**Supplemental Figure 2: The predicted trajectory of combined cognitive function z-score between 2012 and 2020 in older adults by food insufficiency status (n = 4,578).** FS, food sufficiency; FI, food insufficiency. Lines represent the estimated trajectory of combined cognitive score and shaded areas represent the 95%CI. The cognitive decline rates were modeled based on the mixed model with the categorical food insufficiency by year interaction term, adjusting for baseline age, sex, race/ethnicity, education levels, income quintiles, and time-varying variables collected annually for marital status, BMI, depression score, anxiety score, diagnosed status for hypertension, diabetes, heart disease, and heart attack or myocardial infarction. Additionally, the baseline combined cognitive function z-score was adjusted. P-interaction was tested at a significance level of 0.05.

**
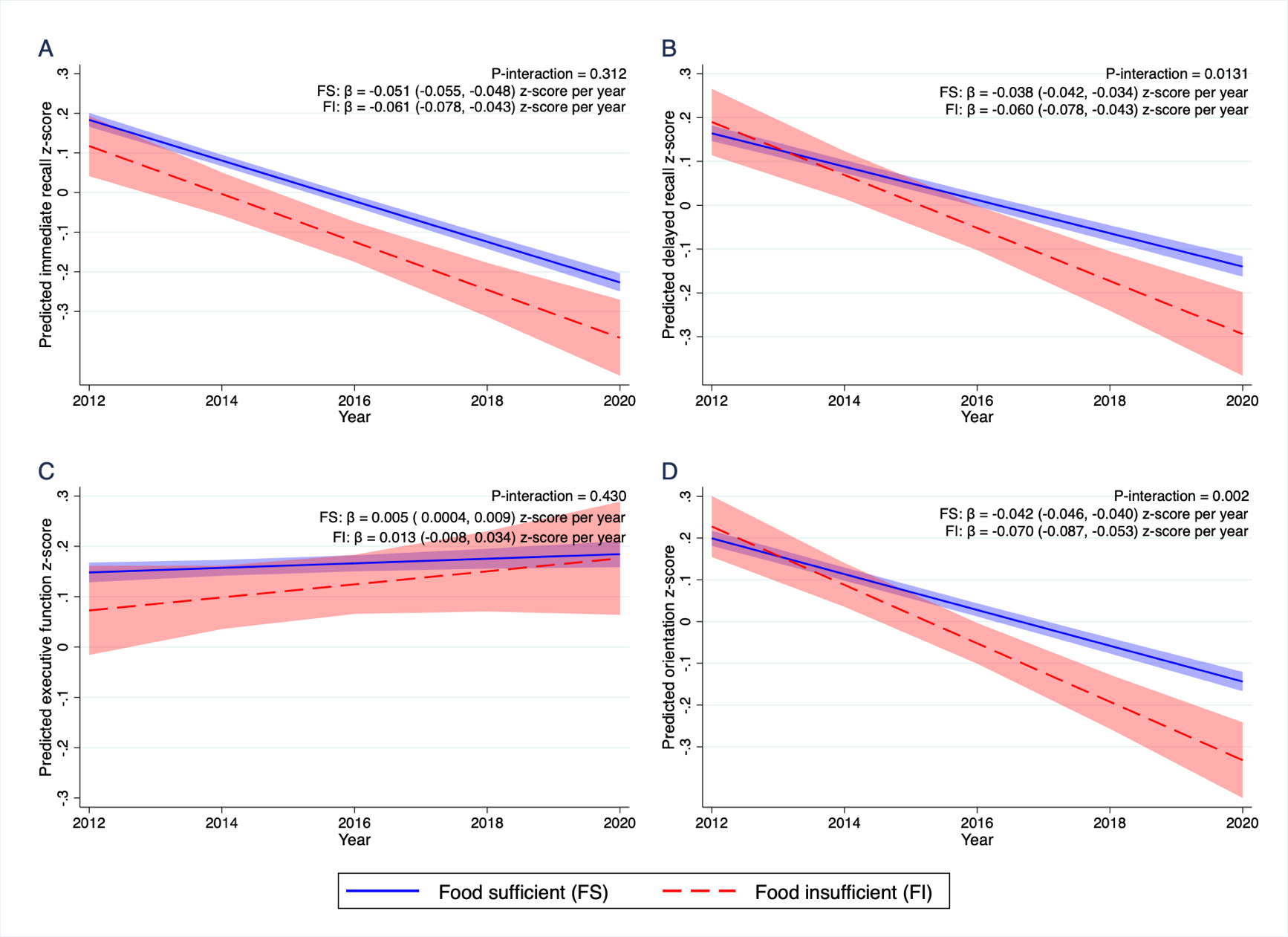
**

**Supplemental Figure 3: The predicted trajectory of (A) immediate recall z-score, (B) delayed recall z-score, (C) executive function z-score, and (D) orientation z-score between 2012 and 2020 in older adults by baseline food insufficiency status (n = 4,578).** FS, food sufficiency; FI, food insufficiency. Lines represent the estimated trajectory of combined cognitive score and shaded areas represent the 95%CI. The cognitive decline rates were modeled based on the mixed model with the categorical food insufficiency by year interaction term, adjusting for baseline age, sex, race/ethnicity, education levels, income quintiles, and time-varying variables collected annually for marital status, BMI, depression score, anxiety score, diagnosed status for hypertension, diabetes, heart disease, and heart attack or myocardial infarction. Additionally, the baseline domain-specific cognitive function z-score was adjusted in each model. P-interaction was tested at a significance level of 0.05.

**
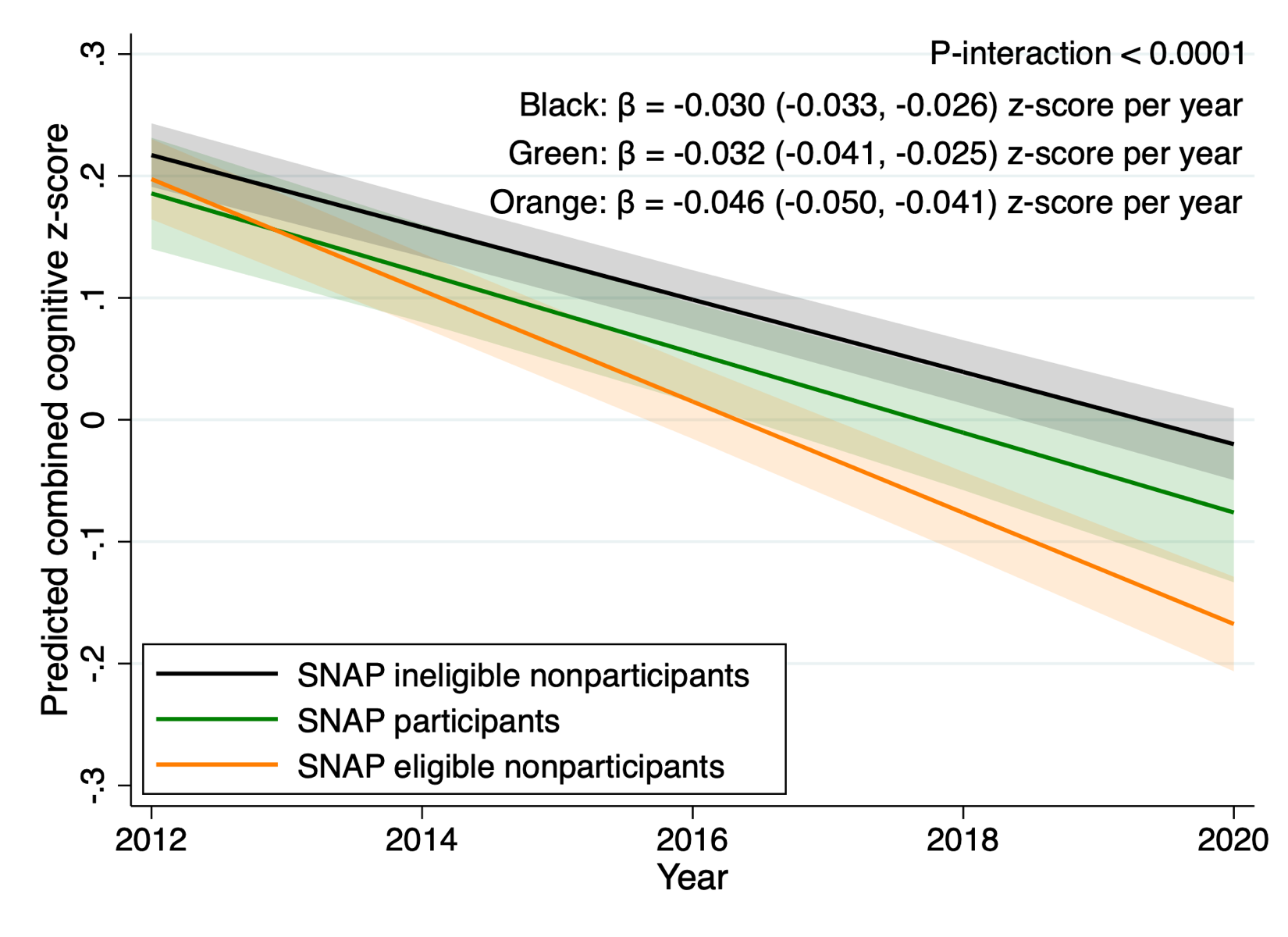
**

**Supplemental Figure 4: The predicted trajectory of combined cognitive function z-score between 2012 and 2020 in older adults by SNAP status (n = 2,832).** SNAP, Supplemental Nutrition Assistance Program. Lines represent the estimated trajectory of combined cognitive score and shaded areas represent the 95%CI. The cognitive decline rates were modeled based on the mixed model with the categorical food insufficiency by year interaction term, adjusting for baseline age, sex, race/ethnicity, education levels, income quintiles, and time-varying variables collected annually for marital status, BMI, depression score, anxiety score, diagnosed status for hypertension, diabetes, heart disease, and heart attack or myocardial infarction. Additionally, the baseline combined cognitive function z-score was adjusted. P-interaction was tested at a significance level of 0.05.

**
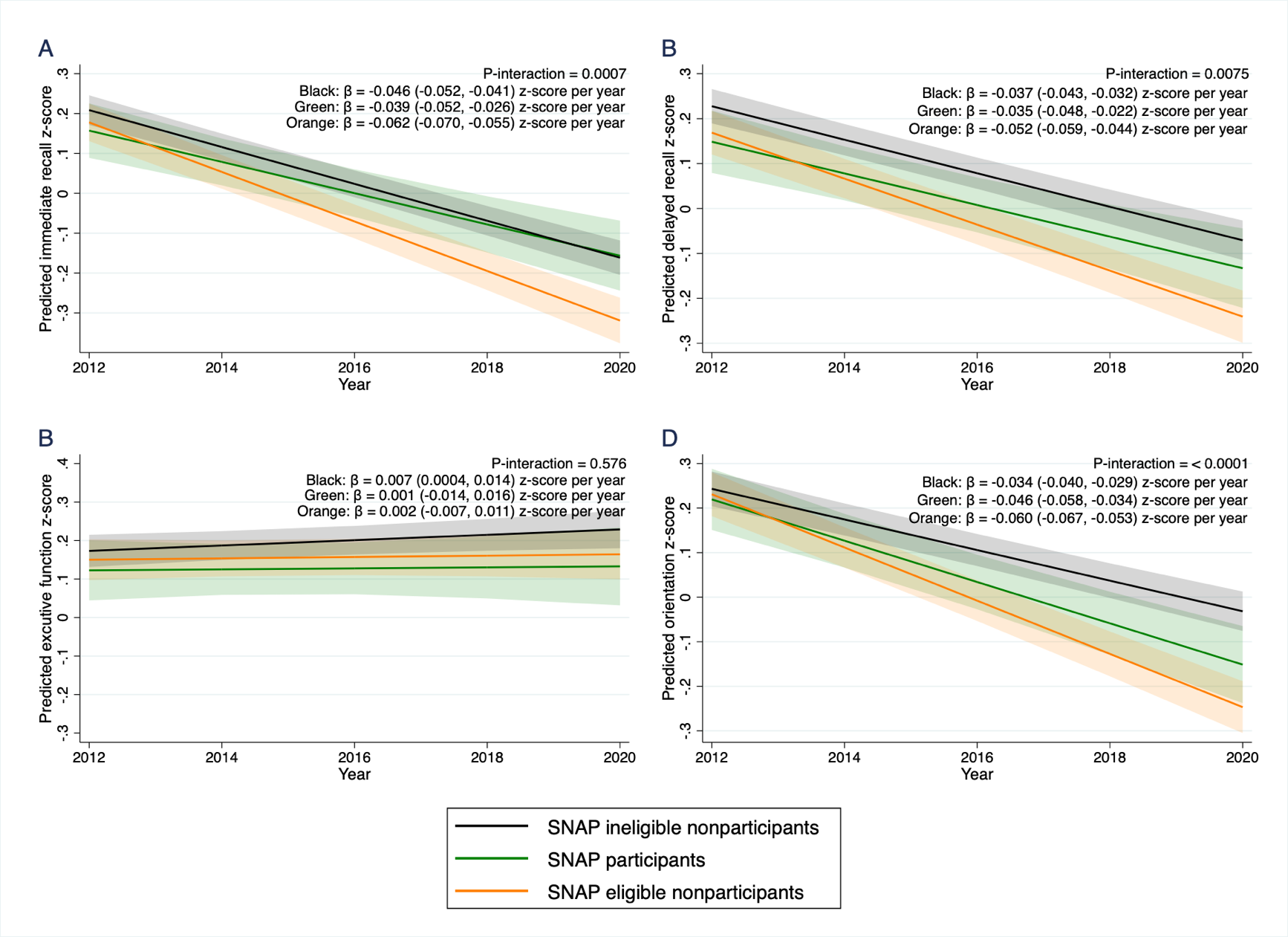
**

**Supplemental Figure 5: The predicted trajectory of (A) immediate recall z-score, (B) delayed recall z-score, (C) executive function z-score, and (D) orientation z-score between 2012 and 2020 in older adults by SNAP status (n = 2,832).** SNAP, Supplemental Nutrition Assistance Program. Lines represent the estimated trajectory of combined cognitive score and shaded areas represent the 95%CI. The cognitive decline rates were modeled based on the mixed model with the categorical food insufficiency by year interaction term, adjusting for baseline age, sex, race/ethnicity, education levels, income quintiles, and time-varying variables collected annually for marital status, BMI, depression score, anxiety score, diagnosed status for hypertension, diabetes, heart disease, and heart attack or myocardial infarction. Additionally, the baseline domain-specific cognitive function z-score was adjusted in each model. P-interaction was tested at a significance level of 0.05.

**
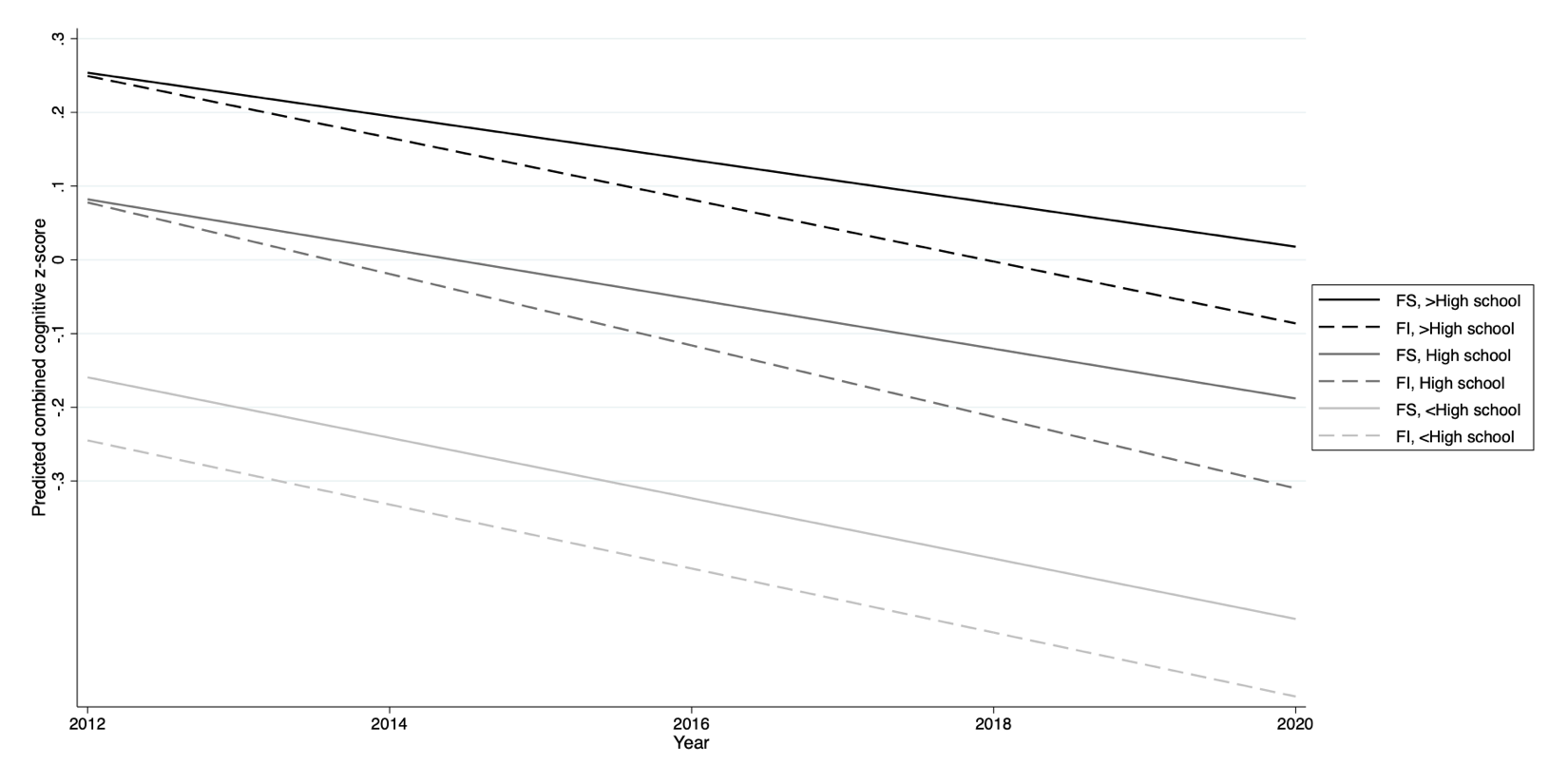
**

**Supplemental Figure 6: The predicted trajectory of combined cognitive function z-score between 2012 and 2020 in older adults by food insufficiency status and education level (n = 4,578).** FS, food sufficiency; FI, food insufficiency. Lines represent the estimated trajectory of combined cognitive score. The cognitive decline rates were modeled based on the mixed model with the three-way interaction term between categorical food insufficiency, year, and baseline education level, adjusting for baseline age, sex, race/ethnicity, education levels, income quintiles, and time-varying variables collected annually for marital status, BMI, depression score, anxiety score, diagnosed status for hypertension, diabetes, heart disease, and heart attack or myocardial infarction. P-interaction = 0.695.
